# Acute Effect of Short-Term Benzocaine Anesthesia on the Skin Mucus Microbiome of Atlantic salmon (*Salmo salar*)

**DOI:** 10.3390/ani15111566

**Published:** 2025-05-27

**Authors:** Patrícia Martins, Tânia Pimentel, Nuno Ribeiro, Ricardo Calado

**Affiliations:** 1 ECOMARE & CESAM (Centre for Environmental and Marine Studies), Department of Biology, University of Aveiro, Campus Universitário de Santiago, 3810-193 Aveiro, Portugal; trpimentel@ua.pt; 2 MVAQUA, Av. Parque de Campismo, Lote 24, Fração C, 3840-264 Vagos, Portugal; nunoribeiro.mvaqua@gmail.com

**Keywords:** aquaculture, RAS, fish skin mucus, bacteria, benzocaine

## Abstract

Routine aquaculture practices such as capture, transportation, and handling can disrupt the relationship between commensal and opportunistic bacteria in the fish skin microbiome. Anesthetic baths are a common welfare practice in aquaculture to reduce stress during handling. However, to date, no studies assessed the effect of anesthetics on bacterial communities in fish skin mucus. This study is the first to evaluate the influence of benzocaine, a widely used anesthetic, on the skin mucus bacterial microbiome of Atlantic salmon reared in a recirculating aquaculture system (RAS). Using Illumina high-throughput 16S rRNA gene sequencing, we found that bacterial richness and diversity were significantly reduced in skin mucus samples from fish with anesthesia (ANE) when compared with those without anesthesia (CTR). The predominant bacterial classes in both groups were Gammaproteobacteria (54.1–62.6%) and Betaproteobacteria (22.6–22.9%). However, significant dissimilarities in beta diversity were observed between the bacterial community structure of salmon skin mucus samples from ANE and CTR. These findings demonstrate that benzocaine exposure alters skin mucus microbiome of Atlantic salmon potentially leading to dysbiosis. This study also provides baseline information on the bacterial communities of Atlantic salmon skin mucus microbiome in an RAS. As no temporal resampling was performed, the duration and persistence of these changes remain unknown and warrant further investigation.

## Simple Summary

Handling fish in aquaculture disrupts the natural balance of skin bacteria, which play a crucial role in disease prevention. Anesthetic baths, used to reduce stress during handling, may further disturb this balance. This study revealed that benzocaine, used as an anesthetic bath, alters the skin mucus bacterial community structure of Atlantic salmon, potentially leading to dysbiosis (an imbalance in bacterial composition). Such disruptions could impair host–microbe interactions and increase susceptibility to opportunistic pathogens. These findings highlight the importance of understanding the impacts of anesthetics on fish microbiomes to improve aquaculture practices, promote fish health, and support more sustainable farming systems.

## 1. Introduction

Sustainable aquaculture relies heavily on maintaining the health and welfare of farmed fish. One critical yet often overlooked aspect of fish health is the role of microbial communities inhabiting their skin. Fish skin microbiome plays a vital role in maintaining host health, immunity, and ecological interactions within aquatic environments [1]. Fishes are always in close contact with their surrounding water, which naturally host a wide diversity of microorganisms [2,3]. As the fish skin is the interface between the organism and its surrounding environment, it plays a paramount role in its protection. In fact, the fish skin mucus is a primary physical and biochemical barrier, which acts as a first line of defence against pathogens that naturally occur in aquatic environments [4,5]. Overall fish health largely depends on the maintenance of a suitable balance between commensal and opportunistic bacteria in fish skin mucus [6,7]. However, in aquaculture, fish are subjected to various stressors and handling practices which may, at times, disrupt the balance of these microbial communities. Therefore, comprehending the dynamics of the fish skin microbiome and its response to environmental perturbations is essential to promote fish welfare, manage disease, and ensure a sustainable production of healthy high-quality fish [8,9].

Anesthetic baths are commonly employed in aquaculture to safely handle fish during multiple procedures, such as grading, tagging, transportation, and when performing medical treatments. These baths require target fish to be immersed in a diluted solution of anesthetic agent(s) for a given time frame, in order to induce a state of sedation or anesthesia that may minimize stress and facilitate the target procedure that needs to be performed with the fish [10,11]. Stress in fish can lead to multiple physiological responses, such as elevated levels of cortisol, compromised immune function, and decreased growth rates [12,13]. Several studies have already focused on the physiological effects of anesthetic baths in fish, such as stress responses [14], shifts in metabolic parameters [15], and impacts on respiratory function [16]. While these studies provide valuable insights into the safety and efficacy of anesthetic agents, the potential impact of these chemicals on the skin microbiome of cultured fish, particularly economically important species such as Atlantic salmon (*Salmo salar*), remains poorly understood.

Atlantic salmon (*Salmo salar*) represent a cornerstone species in global aquaculture, contributing significantly to seafood production and economic livelihoods. This species is among the most intensively farmed in aquaculture, with a global production surpassing 27,000 thousand tonnes in 2020 [17]. Farmed Atlantic salmon accounts for over 32% of all marine and coastal finfish farming, making it one of the most lucrative and technologically advanced sectors within the global fish production industry [17,18]. As aquaculture intensifies to meet growing demand, the use of anesthetic baths becomes increasingly prevalent for routine tasks in fish farms and when performing therapeutic interventions. Yet, the consequences of anesthetic agents on the fish skin microbiome composition and function remain largely unexplored, raising concerns about potential disruptions of host–microbe interactions and susceptibility to opportunistic pathogens.

Traditional culture-based methods and low-resolution molecular techniques often provided an incomplete and potentially biased perspective on microbial diversity, limiting our understanding of microbial interactions and their functional roles. In contrast, advances in high-throughput sequencing technologies have revolutionized our ability to characterize microbial communities and untangle their roles in host physiology and disease susceptibility, particularly within aquaculture [6,19,20,21].

In this study, we addressed the impact of benzocaine on the skin mucus bacterial microbiome of Atlantic salmon (*Salmo salar*), using a high-throughput sequencing approach. Additionally, we characterized the bacterial communities inhabiting the skin mucus microbiome of Atlantic salmon cultured in a recirculating aquaculture system (RAS) located in the southernmost location of this species natural distribution in the Atlantic. The characterization of Atlantic salmon core skin microbiome was performed using salmon skin mucus samples without anesthesia, which we considered as our control group. Given the intimate association between the skin microbiome and its fish host health, establishing baseline information on bacterial communities is crucial for optimizing aquaculture practices and mitigating potential risks to the health of farmed Atlantic salmon.

## 2. Materials and Methods

### 2.1. Study Site, Animals and Feeding

The present study was performed using an RAS located in northern Portugal (40°38′21.1″ N, 8°43′42.2″ W), the southernmost limit of the natural distribution of Atlantic salmon (*Salmo salar*). Briefly, 1236 post-smolts (average fresh weight: 190 ± 30 g) were purchased from a Norwegian hatchery and live shipped by truck in 4 separated tanks (each one with a 2 m^3^ volume) equipped with individual life-support systems. No mortality was recorded during transportation and fish were stocked at a density of ca. 3.1 kg/m^3^ for approximately 5 months in a 320 m^3^ concrete rectangular tank (14 m × 8 m × 2.5 m). Saltwater was pumped from a coastal lagoon (Mira channel in Ria de Aveiro), being sand-filtered before entering the RAS. The RAS was equipped with two sand-filters (ASTRAL Pool, each one with Ø2 m, 1050 kg of 1–2 mm gravel and 3800 kg of 0.4–0.8 mm sand) and microbiological disinfection was secured by two UV filters with three 300 W lamps in each. Biological filtration was performed using a protein skimmer (volume: 2.5 m^3^; filtration rate 40 m^3^/h) and a fluidized biofilter (volume: 3.5 m^3^; filtration rate 40 m^3^/h) (Figure S1). The recirculation water flow was ~120 m^3^/h and a 10% partial water change was performed each week. Fish were fed *ad-libitum*, three times a day, using two different standard commercial diets (Prime 200 and Prime 500, Skretting) adjusted to different fish weights. Daily feed uptake varied from 1.6 to 1.3% of the total biomass. Atlantic salmon fish feed Prime 200 was firstly used until the specimens reached ~450 g fresh weight and Prime 500 was used subsequently, until the sampling moment. The analytical composition of fish feed varied from 25 to 32% for lipids, from 45.2 to 41.6% for crude protein, from 5.7 to 4.9% for crude ashes and from 2.1 to 4.5% for crude fiber. Manual feeding was always performed to better assess overall fish health and welfare. No antibiotics or probiotics were used during this trial.

### 2.2. Salmon Skin Mucus Sampling and DNA Extraction

Twenty Atlantic salmon post-smolts (average weight: 595 g) stoked during approximately 5 months under the culture conditions described above, were randomly collected using hand nets. All fish were visually inspected and showed no external signs of disease or abnormalities and thus, were considered healthy. Ten specimens were immersed up to 10 min in an anesthetic bath (~100 L) using 30–40 mg of benzocaine (commercially sold as Aquacen, 200 mg/mL), while the other ten specimens were exposed to the same procedure but using a saltwater bath alone with no anesthetic. The temperature of the bath was approximately 15 °C for both treatments and matched the temperature of the aquaculture system to minimize thermal stress. Benzocaine was chosen as the anesthetic bath due to its wide use by the aquaculture industry, availability and easiness of acquisition, in accordance with Regulation (EU) No 37/2010). Each sampled fish was placed individually in a separate sterile plastic bag (310 × 500 mm) for approximately 60 s, the time strictly necessary to collect enough skin mucus for further analysis. Following this procedure, all sampled fish were released alive to the production tank, with no mortality being recorded post sampling. All procedures employed are considered ethically accepted and safeguard welfare and best practices during fish handling.

Plastic bags containing samples of skin mucus from fish exposed to anesthesia (ANE) and control fish that were not exposed to anesthesia (CTR) were sealed and immediately transported to the laboratory using a refrigerated polystyrene box. Mucus samples (500 mg each) were collected from the mucus released by the sampled fish that accumulated in the sterilized plastic bags. The mucus was carefully retrieved using a micropipette and transferred into microcentrifuge tubes containing 1 mL of PBS (Phosphate-Saline Solution at pH 7.4) [22]. Samples were centrifuged at 16,000× *g* at 4 °C for 16 min to pellet microbial biomass. After decanting the supernatant, microbial biomass pellets were stored immediately at −20 °C until DNA extraction. Total community DNA (TC) was extracted from skin mucus samples of fish without anesthesia (10 replicates) and from skin mucus samples of fish exposed to anesthesia (10 replicates) using the FastDNA^®^ SPIN soil Kit (MPbiomedicals, Solon, OH, USA) following the manufacturer’s instructions.

### 2.3. Water Sampling and DNA Extraction

Water samples (five replicates) were also collected from two sampling compartments: (1) the supply pipeline of inflowing water (collecting water from the coastal lagoon) (Sup) and (2) the biofilter tank (Bio). Ammonium (NH_3_–N), nitrites (NO^2−^ N), nitrates (N NO^3−^), and phosphates (PO_4_–P) were determined following the 8507, 8016, and 8155 methods described in the Hach Spectrophotometer DR 2800 (Hach, Loveland, CO, USA) standard analytical procedures and according to EPA Method 300.1 and 351.2. Conventional physical-chemical parameters such as, temperature, pH, dissolved oxygen (DO), oxi-redox potential, and salinity were recorded daily at approximately 11 a.m. using specific probes (Hanna HI8424 and HI9147, Hanna Instruments—Woonsocket, RI, USA), which were calibrated regularly following the manufacturer’s instructions.

For bacterial community analysis, water samples were filtered (250 mL) through 0.2 µm pore polycarbonate membranes (Poretics, Livermore, CA, USA) and total community (TC) DNA extraction was performed directly on the filter using an E.Z.N.A Soil DNA Extraction kit (Omega Bio-Tek, Norcross, GA, USA) following the manufacturer’s instructions.

### 2.4. Next-Generation Sequencing Analysis

The V4 hypervariable region of the 16S rRNA gene amplicons were amplified using PCR primers 515F (5′-GTGCCAGCMGCCGCGGTAA-3′) and 806R (5′-GGACTACHVHHHTWTCTAAT-3′) [23] with a barcode on the forward primer [24].

A 30 cycle PCR assay using the HotStarTaq Plus Master Mix Kit (Qiagen, Germantown, MD, USA) was performed under the following conditions: 94 °C for 3 min, followed by 30 cycles of 94 °C for 30 s, 53 °C for 40 s and 72 °C for 1 min, and a final elongation step at 72 °C for 5 min. After amplification, PCR products were checked in 2% agarose gel to determine the success of amplification and the relative intensity of bands.

Multiple samples were pooled together in equal proportions based on their molecular weight and DNA concentrations, which were quantified using a Qubit fluorometer (Thermo Fisher Scientific, Waltham, MA, USA). Pooled samples were purified using calibrated Ampure XP beads AMPure XP beads (Beckman Coulter, Brea, CA, USA), with pooled samples and purified PCR product being used to prepare an Illumina DNA library. Sequencing was performed at MR DNA (www.mrdnalab.com, Shallowater, TX, USA) using Illumina MiSeq (Illumina, San Diego, CA, USA) following the manufacturer’s instructions. Sequence data were processed using MR DNA analysis pipeline (MR DNA, Shallowater, TX, USA). Briefly, sequences were joined, depleted of barcodes and then sequences < 150 bp or with ambiguous base calls were removed. Operational taxonomic units (OTUs) were generated after denoising sequences and filtering chimeras. OTUs were defined by clustering at 3% divergence (97% similarity) and final OTUs were taxonomically classified using BLASTn v2.10.1 against a curated database derived from the Ribosomal Database Project (RDPII) [25] and NCBI (www.ncbi.nlm.nih.gov, accessed on 5 July 2020); see Supplementary Table S1 for the complete OTU dataset. The reference database was curated by integrating high-quality 16S rRNA gene sequences from both the NCBI nucleotide database and the RDP. NCBI-derived sequences were further filtered for quality and trimmed to match the target amplicon region, ensuring consistency and accuracy in taxonomic classification via BLASTn.

### 2.5. Data Analyses

An OTU table containing the raw abundance of all operational taxonomic units (OTUs) of skin mucus samples with anesthesia (ANE) and without anesthesia (CTR) was imported to R. Singletons were removed from the analysis to minimize possible problems with sequencing errors associated with Illumina. The table was log_10_(*x* + 1) transformed and a distance matrix was constructed with the Bray Curtis similarity coefficient using the vegdist() function in the vegan package in R (version 2.11.1; http://www.r-project.org/).

Beta diversity analysis was calculated based on Bray Curtis similarity distances and visualised using principal coordinates analysis (PCO) with the cmdscale() function in R.

Alpha bacterial diversity was assessed by calculating the OTUs recorded, Chao1 index, and Pielou’s evenness index. Both indexes were calculated with the diversity() function in the vegan package in R. Differences in microbial alpha diversity were assessed using the Wilcoxon–Mann–Whitney after rejecting normality using the Shapiro’s test and homogeneity of variance using the Bartlett’s test. Variation in microbial beta diversity and taxa composition were assessed using PRIMER v6 with the PERMANOVA+ add-on software.

To determine the distribution of OTUs in salmon skin mucus samples of fish exposed and not exposed to anesthesia, a Venn diagram was assembled using the venn() function in the gplots package in R. OTUs with more than 50,000 sequence reads were selected and used to assess the most dominant taxonomic groups.

Similarity percentage analysis (SIMPER) was used to reveal the percentage contributions of each OTU to the average dissimilarity between salmon skin mucus samples with and without anesthesia. SIMPER was constructed based on Bray–Curtis distance metrics using PRIMER v6.

To assess the Atlantic salmon core microbiome, we used the skin mucus samples collected from fish that were not exposed to anesthesia, which we considered as our control group. This choice aimed to prevent any potential shifts in the natural bacterial composition of the skin mucus caused by the anesthetic bath. Therefore, whenever we mention skin mucus samples without specifying exposure to anesthesia, we are always referring to samples obtained from fish not exposed to the anesthetic bath.

The microbiome of salmon skin mucus samples (without anesthesia) was compared with the water samples collected from the RAS. Data were assembled into an OTU table and uploaded to R. Variation in OTU composition was visualized using principal coordinates analysis (PCO) following the same method mentioned above. Differences in the bacterial composition of salmon skin mucus associated bacteria and water samples were tested using PERMANOVA. Alpha and beta diversity analysis were assessed using the same methods described above. To investigate the most dominant taxonomic groups, only OTUs with more than 20,000 sequence reads were used. Variation in the relative abundance of the most abundant bacterial OTUs was assessed using bar plot graphs and generated with the barplot2() function in the R.

The core microbiome of salmon skin mucus was assessed using skin mucus samples without anesthesia by selecting the OTUs that were present simultaneously in all individuals.

To assess the presence of potential pathogenic species, OTUs assigned to genera known to be pathogens of Atlantic salmon [26] were selected and investigated. A BLAST search (http://www.ncbi.nlm.nih.gov/) was used to obtain the closest relatives of selected OTUs (pathogens and abundant taxa).

All DNA sequences generated in this study were submitted to the NCBI SRA: Accession number PRJNA1102691.

## 3. Results and Discussion

### 3.1. Effect of Anesthesia in the Skin Mucus Microbiome of Atlantic salmon

#### 3.1.1. Diversity and Community Structure of Bacterial Composition

After quality filtering, 2,831,388 sequence reads were detected and assigned to 1962 OTUs. Fish skin mucus samples of specimens exposed to anesthesia displayed 949,138 reads, while those from fish that were not exposed to anesthesia presented 1,882,250 reads. There was a significant variation in the number of reads between skin mucus samples originating from fish exposed (ANE) or non-exposed to anesthesia (CTR) (PERMANOVA: Pseudo-F = 9.458, *p* = 0.007). The number of OTUs varied from 1581 OTUs in skin mucus samples from fish with anesthesia and 1690 OTUs in fish without anesthesia (Table S1).

**Figure 1:**
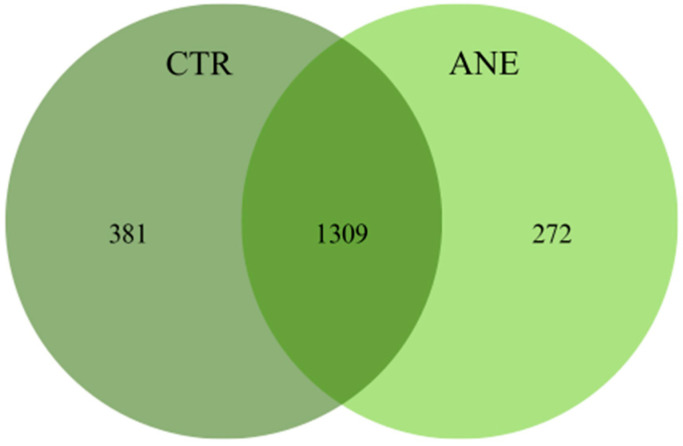
Venn diagram showing the number of bacterial OTUs shared between salmon skin mucus with (ANE) and without anesthesia (CTR).

Shared and unique OTUs between skin mucus samples of both treatments can be observed in the Venn diagram (Figure 1). Skin mucus samples without anesthesia displayed the highest number of unique OTUs, 381 OTUs, while mucus samples with anesthesia displayed the lowest number, only 272 OTUs. Concerning shared OTUs, skin mucus samples of Atlantic salmon shared a total of 1309 OTUs.

**Figure 2:**
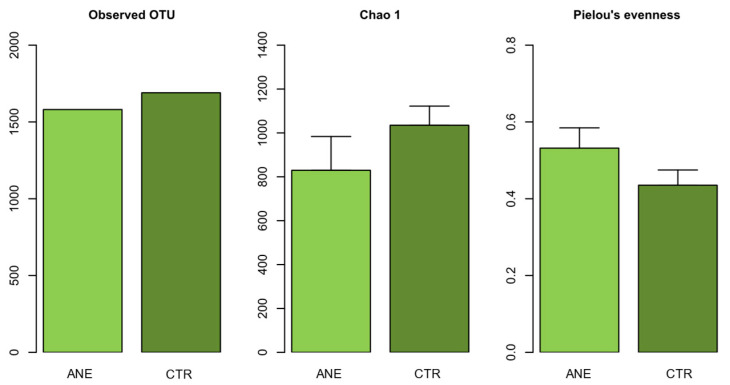
Bacterial α-diversity (Observed OTUs, Chao1 index and Pielou’s evenness index) in the salmon skin samples with (ANE) and without (CTR).

In terms of bacterial community diversity, our results revealed that richness (Observed OTUs) and diversity (Chao1) were lower in skin mucus samples from fish with anesthesia. Bacterial communities in skin mucus samples of fish with anesthesia were more even (Figure 2). Significant differences were found in Chao1 index (Wilcoxon: *p* = 0.019) and Pielou’s evenness (Wilcoxon: *p* = 0.004) (Figure 2). The observed reduction in richness (Chao1), alongside the increase in evennessevenness, in salmon skin mucus samples from fish exposed to anesthesia suggests that additional taxa were present, but their relative abundances were unevenly distributed. These shifts in evenness could be attributed to stress-induced microbial shifts, where certain taxa proliferate while others decline, or even disappear from the community, as a response to exposure to anesthesia. The PCO ordination analysis showed a pronounced separation between bacterial communities from skin mucus samples from fish with anesthesia and those without anesthesia (Figure S2). Significant dissimilarities were also found in the community structure (beta diversity) between salmon skin mucus samples from fish with (ANE) or without anesthesia (CTR) (PERMANOVA: Pseudo-F = 1.847, *p* = 0.036). These results suggested that the use of anesthesia somehow disturbs bacterial communities associated with salmon skin mucus.

#### 3.1.2. Taxonomic Profiling of Bacterial Composition

The overall taxonomic analyses grouped bacterial sequences into twenty phyla, forty classes, and ninety-six orders.

**Figure 3:**
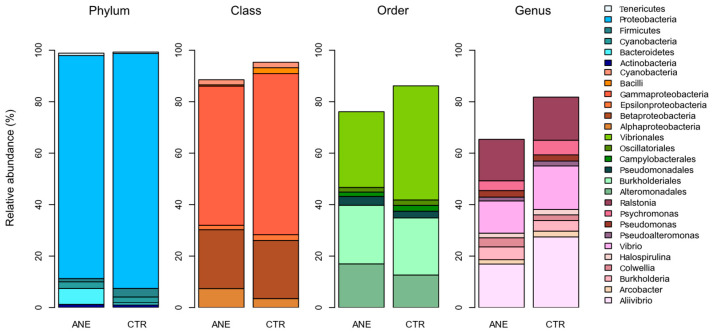
Relative abundance of the most dominant bacterial groups (6 phyla, 6 classes, 6 orders, and 10 genera) identified in the salmon skin mucus samples with (ANE) and without anesthesia (CTR).

Figure 3 highlights the relative abundance of the most dominant bacterial groups (≥50,000 reads) found in fish skin mucus samples. *Proteobacteria* was the most abundant phylum, presenting a relative abundance ranging between 86 and 91%. Phylum *Proteobacteria* consists of a diverse group of bacteria recovered from different hosts and environments. This phylum is also the most common one reported in fish skin mucus microbiome [27,28,29].

The most dominant classes detected in skin mucus communities from fish with or without anesthesia, respectively, were *Gammaproteobacteria* (ranging between 54.1 and 62.6%) and *Betaproteobacteria* (ranging between 22.9 and 22.6%). Concerning orders, the most dominant ones in skin mucus communities from fish with or without anesthesia, respectively, were *Vibrionales* (ranging between 29.4 and 44.3%), *Burkholderiales* (ranging between 22.8 and 22.2%), and *Alteromonadales* (raging between 17.0 and 12.7%). Order *Burkholderiales* had already been previously reported in the skin microbiome of other fish species [30,31]. At genus level, skin mucus communities from fish with or without anesthesia, respectively, were mainly composed by *Ralstonia* (ranging between 16.1 and 16.7%), *Vibrio* (ranging between 12.5 and 16.9%), and *Aliivibrio* (ranging between 16.9 and 27.5%); it is worth highlighting that *Aliivibrio* was the most abundant bacterial genus recorded.

Overall, while salmon skin mucus with and without anesthesia shared common genera, the structure of bacterial communities from skin mucus of fish exposed to anesthesia (ANE) was significantly different from that of conspecifics that were not anesthetized (CTR) (PERMANOVA: Pseudo-F = 6.647, *p* = 0.018). The structure of bacterial communities is known to shift as a response to a given stimulus [32,33], thus suggesting that the microbiome of salmon skin mucus can be affected by benzocaine when an anesthetic bath is applied to a fish being handled.

An in-depth bacterial composition analysis of the skin mucus samples detected six dominant OTUs (≥50,000 reads): OTUs 1 (*Vibrio*), 3097 (*Aliivibrio*), 3110 (*Vibrio*), 2 (*Ralstonia*), 4 (*Burkholderia*), and 427 (*Psychromonas*). Significant differences were detected in the relative abundance of the majority of the most abundant OTUs, except for OTUs 4 and 3110 (Table S2). OTU 3097 was the most abundant OTU with 721,685 sequence reads. According to BLAST this OTU was related to *Aliivibrio wodanis* [GenBank accession number (acc.) JQ361731] isolated from *Oncorhynchus mykiss* (Table S3). This *Aliivibrio* species is considered as a potential pathogen because it is often associated with “winter ulcer” disease in Atlantic salmon [34,35]. However, Klakegg et al. (2020) reported the use of *Aliivibrio* strains as potential probiotic agents to improve the health of farmed fish strains [36].

OTU 2, assigned to the genus *Ralstonia*, was also highly abundant, with a total of 308,514 sequence reads, accounting for 90,921 reads in salmon skin mucus with anesthesia and 217,593 reads in salmon skin mucus without anesthesia. Members of this genus are commonly associated with fish and have been reported as part of the healthy microbiome of Atlantic salmon [37,38]. It has been suggested that these organisms may exhibit antimicrobial activity or contribute to the biosynthesis of bioactive compounds, which could confer beneficial effects on the host [39].

OTU 427, assigned to the genus *Psychromonas*, exhibited a similar trend to OTU 2, with higher abundances in salmon skin mucus samples without anesthesia. Although the ecological role of *Psychromonas* in the marine environment remains not fully understood, it has been reported as a prevalent bacterial genus in the microbiomes of various fish species [40,41].

In order to assess the contribution of each OTU to the dissimilarities recorded in the skin mucus of fish with or without anesthesia, similarity percentages (SIMPER) were determined, with these highlighting five OTUs: 1, 2, 427, 3097, and 3110 (Table 1). OTU 3097 was the OTU that contributed the most (23.7%) for the differences recorded. This OTU was the most dominant one detected in our data and according to BLAST, was related to *Vibrio wodanis.* SIMPER also revealed that OTUs 3110 and 2 contributed with 10.54% and 9.64%, respectively, to the average dissimilarity between skin mucus samples of fish with and without anesthesia. These OTUs were identified as *Vibrio* and *Ralstonia*, respectively, and were also dominant OTUs. Together, OTUs 3097, 3110, and 2 contributed with nearly 44% of all differences recorded between the bacterial communities present in salmon skin mucus samples.

It is important to acknowledge that our study design did not include temporal resampling to evaluate the persistence or reversibility of the observed microbiome shifts following anesthesia. As such, it remains unclear whether the changes in taxonomic composition and diversity represent transient fluctuations or longer-term dysbiosis. Despite these limitations, our findings contribute novel insights into a relatively unexplored area. To the authors best knowledge, to date, there is no information on the potential effect of benzocaine anesthetic baths on fish skin mucus associated microbial communities. However, there are several studies addressing the role of local anesthetics as antimicrobial agents, but always framed under a human health perspective and mostly focusing in lidocaine [42]. Morrow and Berry (1988) studied the antimicrobial properties of benzocaine as a liquid topical anesthetic used in dentistry [43]. In this study, the authors assessed the antimicrobial activity of benzocaine (20%) against several microorganisms commonly found within the oral cavity for 1 min and 2 h [43]. A significant reduction was observed in the growth of all the species investigated when compared with a negative control, for the two-time frames investigated. The results reported by Morrow and Berry (1988) are in line with our data, suggesting that the use of an anesthetic bath does change bacterial communities, with our study showcasing those changes associated with the skin mucus of Atlantic salmon [43].

In fish, skin mucus represents the first barrier against pathogenic organisms, preventing the development of diseases [44]. Fish skin microbiome is colonized by different bacterial species, with their capacity to maintain a healthy balance between commensal and opportunistic bacteria being paramount to ensure synergistic relationships and overall fish health [45]. Nonetheless, it is well documented that aquaculture practices (such as netting, grading, sorting, and transport) can affect the balance of fish skin microbial communities [28]. Therefore, if one highlights that some of these practices commonly require the use of anesthesia, it is important to carefully evaluate the potential effects of using an anesthetic bath on the fish skin microbiome, in order to avoid disturbances on their bacterial communities beyond those already promoted by netting, grading, sorting, or transporting fish. It is important to determine beforehand if the use of anesthesia can induce significant microbial unbalances, to the hypothetical point of even causing dysbiosis in the skin microbiome of fish and/or allow the proliferation of opportunistic bacteria.

Moreover, it is important to mention that most studies assessing the skin or gut microbiome of several fish species, employ anesthesia prior to sampling and, in many cases, fish are even euthanized using an anaesthetic overdose [27,28,46,47,48]. Thus, in light of our findings, previous studies should now be interpreted with further caution, as significant shifts in the microbiome reported for fish in these studies may have been involuntarily shaped through anesthesia.

### 3.2. Comparison of Skin Mucus Microbiome of Atlantic salmon and Rearing Water

#### 3.2.1. Overall Assessment of Bacterial Composition

Physical and chemical parameters recorded during sampling are summarized in Table S4. Temperature, salinity, pH, and nitrites were similar in both water compartments. Salinity remained close to 35, and pH varied between 7.6 and 8.2. On the other hand, and as expected, ammonia, nitrate, and phosphate concentrations were lower in samples from inflowing water being pumped from the coastal lagoon.

**Figure 4:**
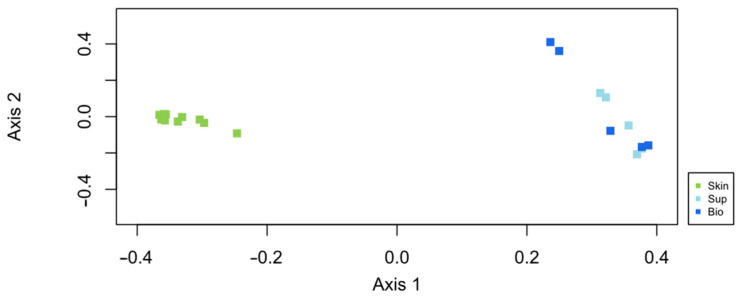
Ordination showing the first two axes of PCO analysis of the bacterial community present: in the salmon skin microbiome (Skin) and in two compartments of the water samples (Sup—Supply water, Bio—Biofilter water).

The PCO ordination analysis (Figure 4) shows a clear separation of two main groups in the ordination representing the water samples and the salmon skin mucus samples. Here, and for all subsequent analyses, skin mucus samples not exposed to anesthesia (CTR) will be used to characterize the bacterial communities associated with Atlantic salmon skin mucus and to compare it with that of surrounding water. The primary axis of variation in the PCO in Figure 4 revealed that bacterial communities present in the water samples and salmon skin mucus samples are clearly different.

Statistically, highly significant differences (PERMANOVA: Pseudo-F = 13.118, *p* < 0.001) were detected in the bacterial composition among groups (water and skin mucus samples). Although we have found differences in some physical and chemical parameters between the water compartments, our results did not reveal distinct microbial communities. In line with these results, the PCO did not show pronounced separation between bacterial communities from different water compartments (biofilter water samples and supply water samples), and statistically no significant differences were observed among the water samples, from a compositional perspective (PERMANOVA: t = 1.209, *p* = 0.188). As such, subsequent statistical analysis performed did not consider the two water compartments individually (5 replicates of water supply + 5 replicates of biofilter water), using instead the 10 water samples as replicates.

Approximately 2,213,372 sequences were recorded and clustered into 1843 bacterial OTUs (after quality filtering) (Table S5). A total of 1522 OTUs were detected in salmon skin mucus samples, while only 727 OTUs were detected in the water samples (Figure S3). Analysis of microbial alpha diversity (Figure S3) showed that the water samples presented a higher diversity when compared to salmon skin mucus samples. Statistically, there are significant differences in alpha diversity between salmon skin samples and the water samples (Chao1 index: Wilcoxon: *p* < 0.001; Pielou’s evenness index: Wilcoxon: *p* = 0.010). Previous studies have also reported highest microbial alfa diversity in the water samples when compared salmon skin mucus and the gut [49].

Although the water samples showed lower microbial richness, OTU abundances were more evenly distributed. The low richness in the water samples is in fact related to the low rare OTUs (OTUs with only 1 or 2 reads) recorded on these samples. On the other hand, beta diversity analysis, calculated based on the Bray–Curtis dissimilarity index and visualized through the PCO ordination analysis (Figure 4), revealed a clear separation of two main groups in the ordination, representing the water samples and the salmon skin mucus samples. As aforementioned, significant differences were detected between the salmon skin mucus microbiome and that of the water samples.

#### 3.2.2. Taxonomic Bacterial Composition

**Figure 5:**
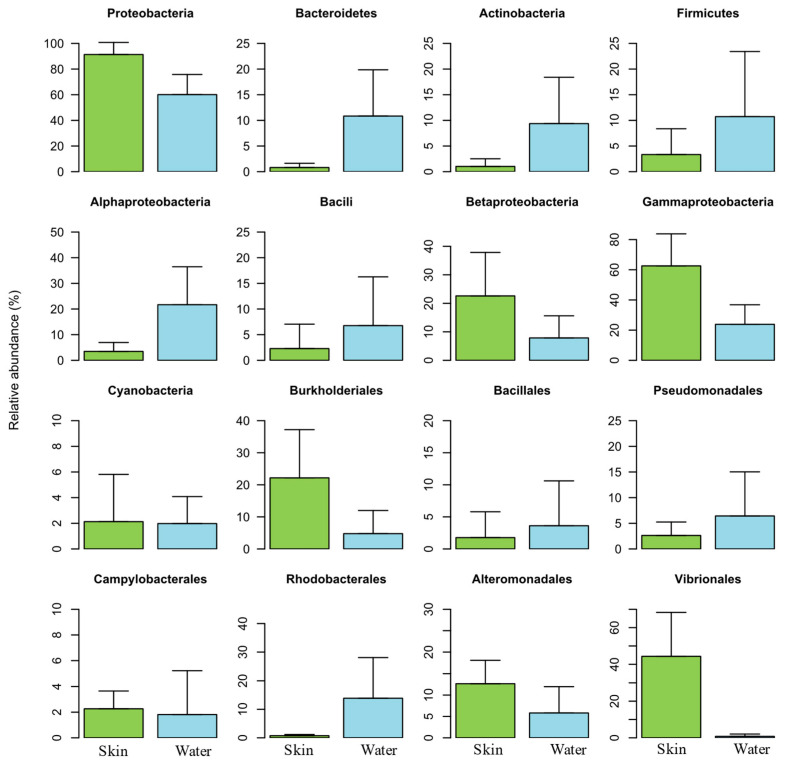
Relative abundance of the most dominant bacterial groups (4 phyla, 5 classes, and 7 orders) in the salmon skin microbiome (Skin) and in the water samples (Water).

The overall taxonomic analyses grouped bacterial sequences into 35 phyla, 84 classes, and 171 orders. Figure 5 highlights the relative abundance of the most dominant bacterial groups. *Proteobacteria* was the most abundant phylum (ranging between 58.1 and 89.2%), followed by *Actinobacteria* (ranging between 1 and 12.7%), *Firmicutes* (ranging between 3.3 and 11.3%), and *Bacteroidetes* (ranging between 0.8 and 11.2%). Phylum *Proteobacteria* was the most dominant in salmon skin mucus samples, while phyla *Actinobacteria*, *Firmicutes* and *Bacteroidetes* were the most dominant in the water samples. A predominance of the phyla *Proteobacteria*, *Actinobacteria*, *Firmicutes*, *Bacteroidetes* has also been previously reported on the skin mucus microbiome of Atlantic salmon, in a study assessing the skin associated microbiota during the transition of individuals of this species from freshwater to seawater [27].

The majority of OTUs classified within the class *Gammaproteobacteria* were classified into the orders *Vibrionales*, *Alteromonadales*, and *Pseudomonadales* (Figure 5). While the orders *Vibrionales* e *Alteromonadales* were dominant in salmon skin mucus samples, the order *Pseudomonadales* was dominant in the water samples. The high abundances of this order in the water samples are related to the family *Moraxellaceae* and specifically the genus *Acinetobacter*. Members of this genus had been already identified as emerging fish pathogens [50].

#### 3.2.3. Composition Analysis of Dominant OTUs in Salmon Skin Mucus

The dominance analysis revealed nine OTUs (≥20,000 sequence reads) in the salmon skin mucus microbiome: OTU 3097 (*Aliivibrio wodanis*), OTU 3110 (*Vibrio*), OTU 2 (*Ralstonia solanacearum*), OTU 427 (*Psychromonas*), OTU 1 (*Vibrio*), OTU 4 (*Burkholderia*), OTU 7 (*Pseudoalteromonas*), OTU 14 (*Paenibacillus*), and OTU 8 (*Pseudomonas*) (Figure S4 and Table S3).

The genera *Vibrio*, *Ralstonia*, *Burkholderia*, *Pseudoalteromonas*, *Psychromonas*, and *Pseudomonas* were previously reported as the most abundant groups in the skin associated microbiome of Atlantic salmon [27,28,51].

*Aliivibrio wodanis*, the most abundant OTU, is frequently isolated (concurrently with *Moritella viscosa*) from farmed Atlantic salmon with winter-ulcer disease [35,52]. However, Karlsen et al. (2014) observed that A. *wodanis* alone can also cause infection in Atlantic salmon, although its colonization requires a predisposed damage in the host area of colonization, a feature which suggests a low virulence [34]. As already referenced, Klakegg et al. (2020) described *A*. *wodanis* as a potential probiotic, namely against *M. viscosa* [36]. The authors revealed an increase in growth and resistance of cultured lumpfish (*Cyclopterus lumpus*) against bacterial diseases after they were exposed to probiotic *A*. *wodanis.* Likewise, members of the genera *Paenibacillus* and *Pseudoalteromonas* have also shown potential probiotic properties against several aquaculture bacterial pathogens [53,54]. According to Aranda et al. (2012), *Pseudoalteromonas* sp. strains can be used as *Vibrio*-biocontrol agents, as they are able to produce a putatively novel class of bacteriostatic compounds [53].

#### 3.2.4. Core Microbiome Composition of Atlantic salmon

Of the 1522 OTUs found in salmon skin mucus samples, only 143 were common to all individuals sampled, thus forming the core microbiome (Table S6). The core skin mucus microbiome was formed by 14 classes, 27 orders, and 28 families that enclosed a total of 65 different bacterial genera. The core skin mucus microbiome of Atlantic salmon is predominantly composed by proteobacterial OTUs. *Proteobacteria* was the most representative phylum in the core microbiome of Atlantic salmon, displaying a relative abundance of 95%. *Gammaproteobacteria* and *Betaproteobacteria* were the most dominant classes featuring a relative abundance of 66% and 24%, respectively. *Proteobacteria* has been reported as the most predominant bacterial phylum in the skin-associated communities of the core microbiome of Atlantic salmon, highlighting their key role in the microbiome of this fish species [49,55].

Although *Vibrionales* (47%) was the most dominant order at the core of salmon skin mucus bacterial composition, orders *Burkholderiales* (24%) and *Alteromonodales* (13%) also showed high relative abundances. At family level, *Vibrionaceae* (47%), *Burkholderiaceae* (23%), and *Psychromonadaceae* (6%) were the ones featuring higher relative abundances in the skin mucus of Atlantic salmon core microbiome. From the 66 genera identified in the core microbiome of Atlantic salmon skin mucus, the most abundant ones were *Aliivibrio* (28%), *Vibrio* (19%), *Ralstonia* (18%), *Psychromonas* (6%), and *Burkholderia* (4%). Genus *Aliivibrio* was established in 2007, with the reclassification of several *Vibrio* species [56]. *Vibrio* spp. are Gram-negative bacteria, that are ubiquitous in marine, estuarine, and freshwater environments. Both genera, *Aliivibrio* and *Vibrio* are considered pathogenic, as they are frequently associated with diseased animals. Several members of *Vibrio* and *Aliivibrio* (*V. anguillarum*, *V. parahaemolyticus*, *V. vulnificus*, *Aliivibrio salmonicida*) are responsible for Vibriosis, one of the most common bacterial diseases in aquaculture systems [57,58,59]. The high pathogenicity of these genera is related to its adhesive capacity to mucosal surfaces, which could explain the high abundance of these taxa in the skin mucus of Atlantic salmon [60,61]. Minniti et al. (2019), in a study assessing the skin–mucus proteome of farmed Atlantic salmon, also recorded genus *Vibrio* as the most abundant bacterial genera in this biological matrix [51]. Other authors have described genus *Vibrio* and *Ralstonia* as the most abundant in the skin mucus microbiome of Atlantic salmon [28,51], findings that are certainly aligned with the ones reported in the present study.

From the ten dominant OTUs found in the skin mucus microbiome of Atlantic salmon, nine were detected in the core microbiome. This finding points out the importance of identifying and understanding the most dominant taxa present on fish skin mucus microbiome, in order to allow the establishment of baseline information (in terms of microbiome composition and structure) that can later be helpful to monitoring the overall health of cultured fish.

Overall, our study recorded several similarities in the core skin mucus microbiome of Atlantic salmon with that reported in previous studies, although it is worth highlighting that despite Atlantic salmon being one of the most important aquaculture species worldwide, there still is a generalized lack of information concerning the skin mucus microbiome of this species.

#### 3.2.5. Bacterial Community Profile of Rearing Water

*Proteobacteria* and *Bacteroidetes* were the main dominating phyla in the water samples analyzed, although *Firmicutes* and *Actinobacteria* also presented high relative abundances (Figure 5). Salmon skin mucus and water samples had similar bacterial compositions at phylum-level, although these displayed different abundances.

Orders *Pseudomonadales*, *Actinomycetales* and *Alteromonadales* were the most abundant ones detected in the water samples (Figure S5).

As above mentioned, *Acinetobacter* was the most abundant genus in the water samples, displaying a relative abundance of 4.8%. Nonetheless, it is worth highlighting that genera *Roseobacter* and *Streptococcus* were also abundant in the water samples analyzed, with a relative abundance of 4.6% and 3%, respectively (Figure S5).

The dominance of genus *Acinetobacter* in the water samples is mostly explained by OTU 34, as this was the most abundant OTU found in the water samples processed, with 12,865 sequence reads. BLAST (http://www.ncbi.nlm.nih.gov/ results related this OTU to *Acinetobacter junii* [GenBank accession number (acc.) MT613873] isolated from a water surface in India. This bacterium was recorded as a nosocomial pathogen and can be found colonizing human and environmental samples [62]. Members of genus *Acinetobacter* had already been identified as emerging fish pathogens, with Malick et al. (2020) having identified *Acinetobacter junii* as the causative agent of a fish disease outbreak in India [50].

Genus *Roseobacter* belongs to the *Roseobacter* clade, one of the major marine bacterial groups, comprising up to 20% of all bacterioplankton communities in the ocean [63], thus it was not a surprise to find that this is also one of the main dominating bacteria detected in the bacterioplankton communities of several aquaculture systems [19,29]. Furthermore, previous studies suggest that the *Roseobacter* clade may play an important role against the development of fish pathogens in aquaculture systems [64,65]. In fact, Martins et al. (2018) suggested that the high abundance of members of the *Roseobacter* clade may play a role in suppressing the development of *Vibrio* lineages in aquaculture systems [66].

OTUs 63 and 169 were the most abundant OTUs in the water samples, being allocated to genus *Streptococcus* (8450 sequence reads) and having been identified as *S. parasanguinis* and *S. thermophillus*, respectively. Although genus *Streptococcus* is mostly associated with fish diseases [67], there are evidence that some species, such as *S. thermophillus*, may also be used as probiotic agent in aquaculture [68].

All of the most abundant genera of bacteria detected in the water samples were also detected in the skin mucus samples of Atlantic salmon, in line with previous reported by Minniti et al. (2017) [28]. Overall, while we have found some differences in the microbial composition between the samples of fish skin mucus and water that were analyzed in our study, our data suggest that culture water shapes the diversity and structure of Atlantic salmon skin mucus microbiome. Approximately 55% (406 OTUs) of the OTUs found in the water samples analyzed were also detected in the skin mucus of sampled fish. These results agree with previous studies that suggest that fish skin has specialized microbial communities that are influenced by surrounding water but are still able to feature distinct bacterial taxa [28,49].

#### 3.2.6. Potential Pathogens Detected in the Skin Mucus Microbiome of Atlantic salmon and in Rearing Water

**Figure 6:**
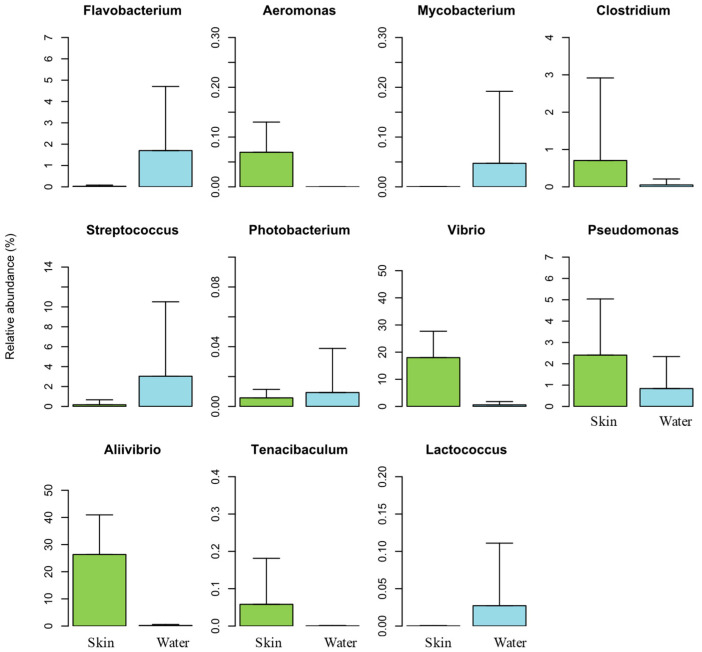
Relative abundance of potential pathogenic genera detected in the salmon skin microbiome (Skin) and in the water samples (Water).

To understand the presence of potential pathogenic bacteria in the skin mucus microbiome of Atlantic salmon and surrounding water, the relative abundance of OTUs belonging to genera known to be potential fish pathogens was investigated. From the 16 potential pathogenic genera listed in the scientific literature [69], 11 were detected in our samples (Figure 6).

*Aliivibrio* and *Vibrio* were the most abundant potential pathogenic genera found in our samples, with a relative abundance of 26% and 17% (552,762 and 374,145 sequence reads) in fish skin mucus samples and only 0.2% and 0.5% (591 and 1660 sequence reads) in the water samples, respectively. As already mentioned, the most abundant OTU detected in our data was OTU 3097 (*Aliivibrio wodanis*) with 552,593 sequence reads. This OTU alone accounts for almost the total abundance of genus *Aliivibrio* in our work. Despite species within this genus being considered as potential pathogens, there are evidence that *Aliivibrio* strains, specifically *A. wodanis*, can also be used as probiotic agents in aquaculture [36]. Genera *Aliivibrio* and *Vibrio* were significantly more abundant in samples from salmon skin mucus than in those from culturing seawater, most likely because of their high adhesive ability to mucosal surfaces [60,61].

Genera *Aliivibrio* (26%), *Vibrio* (17%), *Pseudomonas* (2.4%), *Clostridium* (0.7%), and *Aeromonas* (0.07%) showed higher abundances in Atlantic salmon skin mucus samples, while *Streptococcus* (3%), *Flavobacteria* (1.7%), *Mycobacterium* (0.05%), *Lactococcus* (0.02%), and *Photobacterium* (0.009%) presented higher abundances in the water samples (Figure 6). Furthermore, genera *Vibrio*, *Aliivibrio*, *Flavobacterium*, *Mycobacterium*, *Streptococcus*, *Photobacterium*, and *Lactococcus* were significantly more abundant in Atlantic salmon skin mucus samples. These results suggest that this biological matrix in fish is an important reservoir for many of the potential bacterial pathogens surveyed in aquaculture, both in terms of abundance and pathogenic species diversity.

Genera *Renibacterium*, *Nocardia*, *Aerococcus*, *Enterobacterium*, and *Edwardsiella*, which are well known for their pathogenicity in aquaculture, were not detected in any of the samples surveyed in the present work.

Genus *Aeromonas* was the only one detected exclusively in salmon skin mucus samples (3 OTUs), although we did not detect *A. salmonicida*, one of the most important primary pathogens impacting salmonid fish [70]. Likewise, despite the high abundance of *Vibrio* and *Aliivibrio* recorded in our samples, the species *Aliivibrio salmonicida* (former *Vibrio salmonicida*) was not recorded. This bacterium is the causative agent of cold-water vibriosis that causes hemorrhagic septicemia and occurs mostly in farmed Atlantic salmon [71,72].

Apart from *Aliivibrio* and *Vibrio*, all the other potential pathogenic bacterial genera detected were present at a very low abundance. In fact, genera *Photobacterium*, *Mycobacterium*, *Aeromonas*, *Tenacibaculum*, *Lactococcus*, and *Clostridium* all together accounted for less than 1% of the total bacterial community detected both in fish skin mucus and seawater. Despite the presence of potential pathogenic bacterial genera, no diseased fish were registered during the study period.

## 4. Conclusions

In the present study, we assessed the influence of a benzocaine anesthetic bath on the skin mucus microbiome of cultured Atlantic salmon. While previous studies have reported the antimicrobial properties of anesthetic agents, to date this issue has been mostly addressed in the context of human health. Our results revealed that the use of benzocaine as an anesthetic bath impacts the skin mucus microbiome of Atlantic salmon. However, further investigation is certainly needed to better understand the mechanisms behind these shifts, whether the pre-anesthesia fish skin mucus microbiome is re-established post-anesthesia, and what the timeframe for such recovery might be. It is important to note that this study did not include temporal resampling to evaluate the duration or persistence of microbiome changes following anesthesia. Therefore, future research should explore these temporal dynamics and assess the effects of different anesthetic agents and exposure conditions. Moreover, it is also important to determine if similar results to the ones reported in the present study are also observed under different experimental conditions (e.g., when employing different anesthetic agents, using different times of exposure, multiple and/or consecutive exposures, life stages, and fish species). To the best of our knowledge, this is the first study reporting the effect of an anesthetic bath on the skin microbiome of farmed Atlantic salmon, paving the way for a new research line on fish health and welfare.

## Figures and Tables

**Table 1:** Similarity percentage analysis (SIMPER) identifying which OTU contribute to the differences detected in the skin microbiome of samples with (ANE) and without anesthesia (CTR).

OTU	Genus Identification	Average Abundance ANE	Average Abundance CTR	Ind. %	Cum.%
**OTU 3097**	Aliivibrio	16,909.20	55,259.30	23.68	23.68
**OTU 3110**	Vibrio	10,091.20	24,032.70	10.54	34.22
**OTU 2**	Ralstonia	9092.10	21,759.30	9.64	43.86
**OTU 427**	Psychromonas	3147.50	9258.50	4.18	48.04
**OTU 1**	Vibrio	2505.50	8468.00	3.70	51.73
**OTU 4**	Burkholderia	2840.30	4570.80	1.93	53.66
**OTU 14**	Bacillus	2.10	3257.40	1.68	55.34
**OTU 8**	Pseudomonas	2106.20	2732.90	1.67	57.01
**OTU 7**	Pseudoalteromonas	1265.70	3465.60	1.62	58.63
**OTU 30**	Tenacibaculum	2054.60	135.90	1.44	60.07
**OTU 16**	Altererythrobacter	1069.20	1768.30	1.21	61.28
**OTU 32**	Colwellia	2604.90	1908.80	1.20	62.48
**OTU 19**	Massilia	1599.10	984.50	1.08	63.57

## Data Availability

Raw sequence files supporting the results of this article are available in NCBI SRA: Accession number PRJNA1102691.

## Supplementary Materials

The following supporting information can be downloaded at https://www.mdpi.com/article/10.3390/ani15111566/s1, Figure S1: RAS unit description. (a) stocking tank; (b) recirculation water-pumps; (c) protein skimmer; (d) fluidized biofilter; (e) one out of two UV sterilization filter tank; Figure S2: Ordination diagram showing the first two axes of PCO analysis of the bacterial community present in the salmon skin mucus samples (ANE—skin mucus with anesthesia, CTR—skin mucus without anesthesia); Figure S3: Bacterial species α-diversity measures (Observed OTUs, Chao1 index and Pielou’s evenness index) in the salmon skin samples (Skin) and in the water samples (water); Figure S4: Relative abundance of dominant OTUs (≥20,000 sequence reads) in the salmon skin microbiome (Skin); Figure S5: Relative abundance of the four most dominant orders and genera detected in water samples. Skin—salmon skin microbiome; Water—water samples; Table S1: OTU table with taxonomic classification (AN—salmon skin samples with anesthesia; NO—salmon skin samples without) anesthesia); Table S2: PERMANOVA main test (F value and P value) between salmon skin samples with and without anesthesia, for each OTU; Table S3: Taxonomic affiliation of the most abundant OTUs in salmon skin mucus and water samples (≥20,000 sequences) including OTU-numbers (OTU); number of sequence reads; taxonomic assignment; their closest relatives (using Blast); with respective accession number (Acession); sequence identity (Sq ident); and their source; Table S4: Values of temperature, pH, dissolved oxygen (DO), salinity, oxi-redution potential, ammonia, nitrites, nitrates and phosphates in the aquaculture compartments. Sup—Supply water samples; Bio—Biofilter water sample; Table S5: OTU table with taxonomic classification (Skin—salmon skin mucus; Water—water samples); Table S6: OTUs forming the skin core microbiome of Atlantic salmon.
