# Supplementary material for: Acute Effect of Short-Term Benzocaine Anesthesia on the Skin Mucus Microbiome of Atlantic salmon (Salmo salar)
Source: Animals (Basel). 2025 May 27;15(11):1566. doi: 10.3390/ani15111566 (PMC12153927; doi:10.3390/ani15111566)
Supplement: Supplementary file 1 [file animals-15-01566-s001.zip › animals-3435846--Supporting information.pdf]

Supplementary materials

Effect of anesthesia in the skin mucus microbiome of Atlantic salmon (*Salmo salar*)

Patrícia Martins[1*], Tânia Pimentel[1], Nuno Ribeiro[2], Ricardo Calado[1*]

[1] ECOMARE, CESAM (Centre for Environmental and Marine Studies), Department of Biology, University of Aveiro, Campus Universitário de Santiago, 3810-193 Aveiro, Portugal

[2]MVAQUA, Av. Parque de Campismo, Lote 24, Fração C, 3840-264 Gafanha da Boa Hora, Portugal

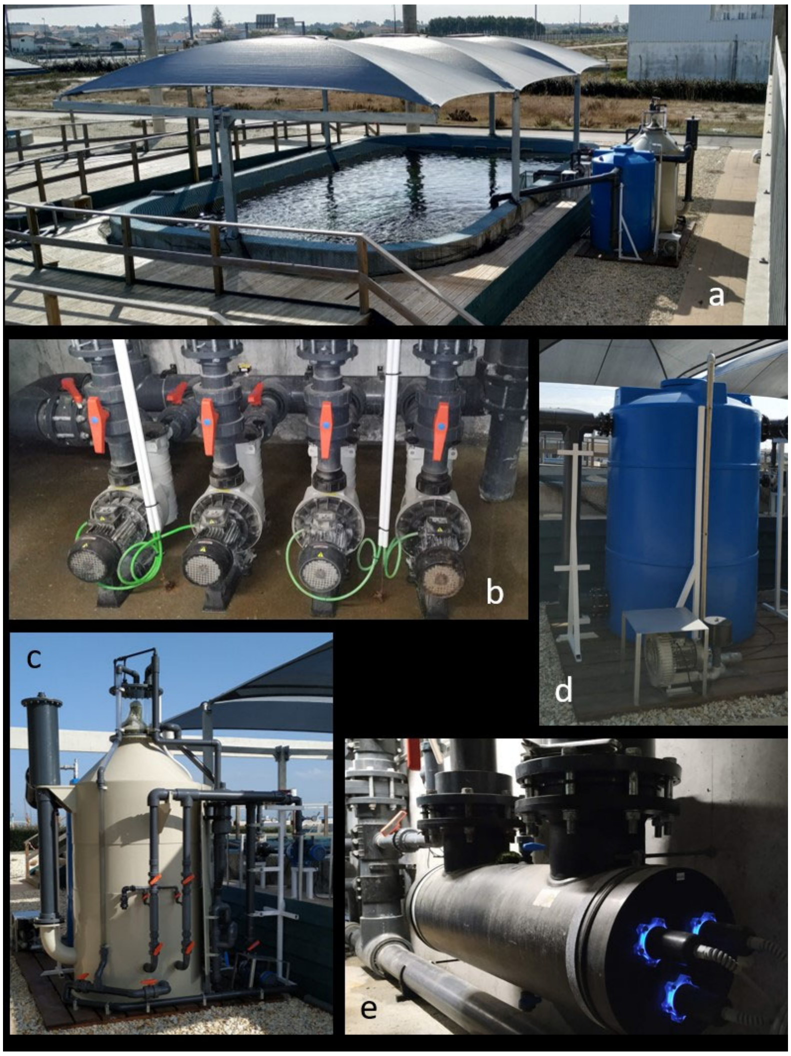

Figure S1 - RAS unit description. a) stocking tank; b) recirculation water-pumps; c) protein skimmer; d) fluidized biofilter; e) one out of two UV sterilization filter tank.

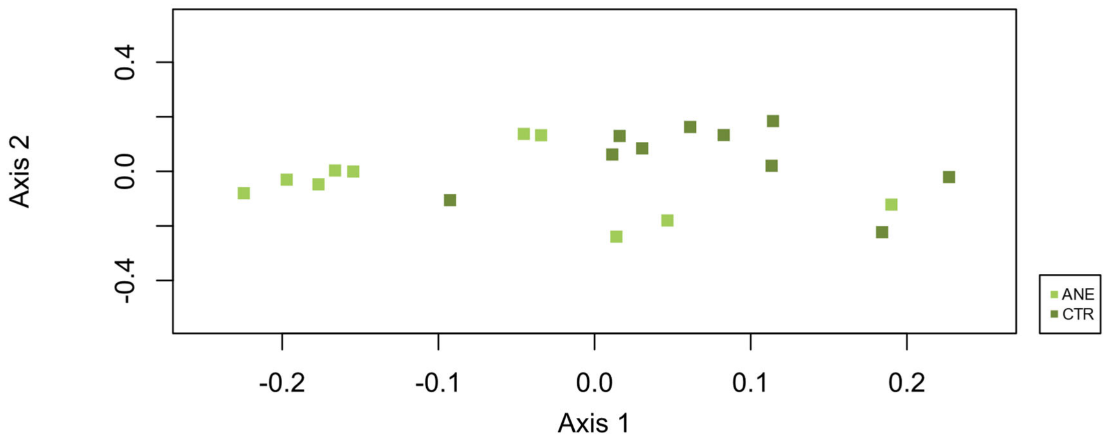

Figure S2 - Ordination diagram showing the first two axes of PCO analysis of the bacterial community present in the salmon skin mucus samples (ANE – skin mucus with anesthesia, CTR – skin mucus without anesthesia).

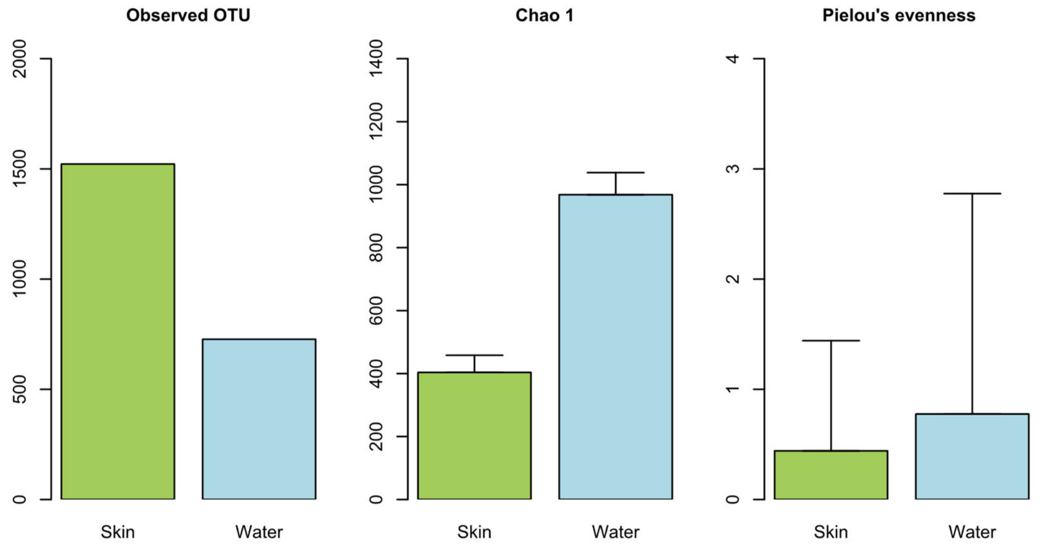

Figure S3 - Bacterial species α-diversity measures (Observed OTUs, Chao1 index and Pielou's evenness index) in the salmon skin samples (Skin) and in the water samples (water).

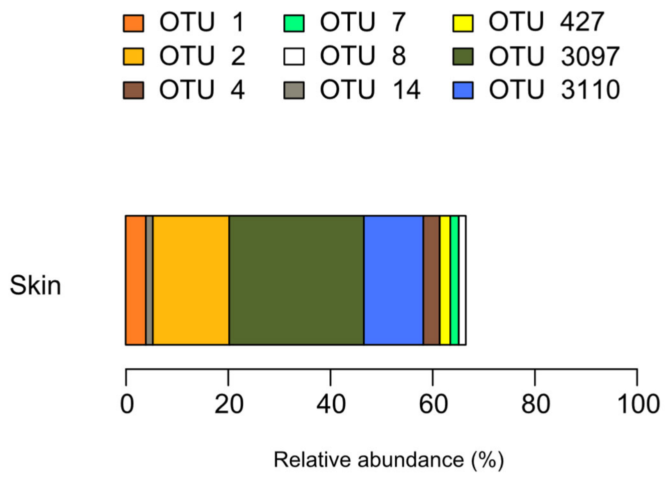

Figure S4 - Relative abundance of dominant OTUs (≥ 20000 sequence reads) in the salmon skin microbiome (Skin).

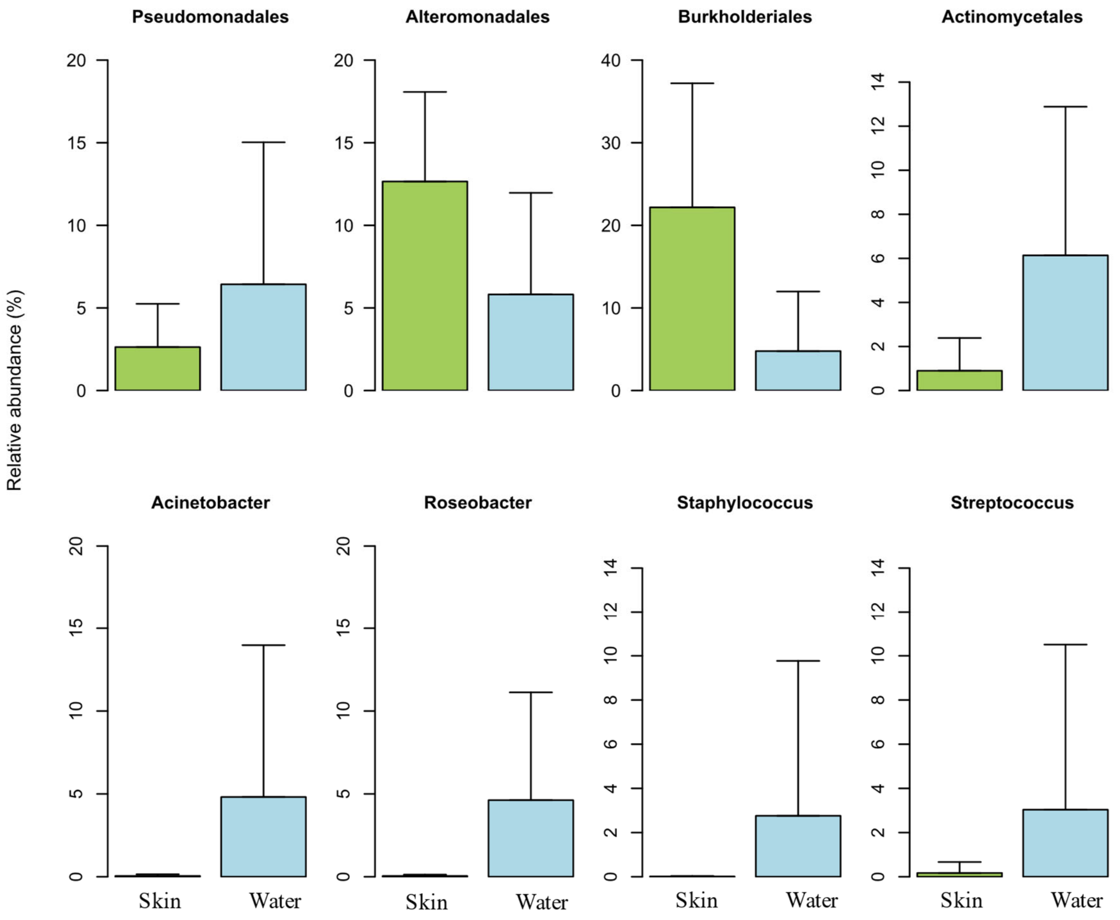

Figure S5 - Relative abundance of the four most dominant orders and genera detected in water samples. Skin - salmon skin microbiome; Water - water samples.
